# Semaglutide reduces murine blood pressure through the vascular smooth muscle GLP-1 receptor

**DOI:** 10.1172/jci.insight.201148

**Published:** 2026-03-03

**Authors:** Kyle D. Medak, Jacqueline A. Koehler, Laurie L. Baggio, Maria J. Gonzalez-Rellan, Chi Kin Wong, Xiemin Cao, Vivikta Rao, Sean Kao, Yu Cui, Jiayi Fu, Easton Liaw, M. Golam Kabir, Jie Zhang, Jin Wei, Daniel J. Drucker

**Affiliations:** 1 Lunenfeld-Tanenbaum Research Institute, Sinai Health System, Toronto, Ontario, Canada.; 2 Section of Nephrology, Department of Medicine, Boston University Chobanian & Avedisian School of Medicine, Boston, Massachusetts, USA.

**Keywords:** Cardiology, Endocrinology, Metabolism, Therapeutics

## Abstract

GLP-1 receptor (GLP-1R) agonists decrease blood glucose and body weight and reduce rates of cardiovascular and renal disease. Although GLP-1R activation lowers blood pressure (BP), the underlying mechanisms remain incompletely understood and have been attributed to weight loss and endothelial cell GLP-1R signaling. Here, we show that GLP-1Rs in vascular smooth muscle cells (VSMCs) are essential for semaglutide-mediated BP reduction in mice. In contrast, GLP-1Rs in Tie2^+^ endothelial or immune cells are not required for semaglutide to lower BP. The VSMC GLP-1R is dispensable for the effects of semaglutide on food intake, body weight, and blood glucose but is required for its actions to increase glomerular filtration rate and promote natriuresis. Systemic semaglutide administration resulted in proteomic changes in the renal artery and kidney in pathways related to platelet aggregation, fibrin clot formation, lipid metabolism, and proapoptotic signaling that are abolished in mice lacking VSMC GLP-1R expression. Moreover, semaglutide directly induced vasorelaxation in preconstricted mesenteric arteries ex vivo. Together, these findings identify VSMCs as a key cellular target linking GLP-1R activation to BP regulation, renal electrolyte excretion, and proteomic changes in renal artery and kidney.

## Abstract 2

These studies identify the vascular smooth muscle GLP-1 receptor as a key cellular target for the actions of GLP-1 medicines to lower blood pressure

## Introduction

GLP-1 receptor agonists (GLP-1RAs) are widely used for the treatment of type 2 diabetes (T2D) and obesity (1). Beyond their metabolic effects, GLP-1RAs such as dulaglutide, liraglutide, and semaglutide reduce the incidence of major adverse cardiovascular events (MACE) (2) and improve outcomes in individuals with heart failure with preserved ejection fraction (3). Additionally, GLP-1RAs reduce albuminuria and slow the rate of estimated glomerular filtration rate (eGFR) decline in patients with T2D (4, 5). Furthermore, semaglutide attenuates the progression of chronic kidney disease and reduces cardiovascular death in patients with T2D and diabetic kidney disease (6), and slows eGFR decline and progression to end stage chronic kidney disease in patients with obesity and atherosclerotic cardiovascular disease (7).

Despite the growing evidence for cardiorenal benefits of GLP-1 medicines, the precise mechanisms underlying these benefits remain incompletely understood. GLP-1 medicines reduce HbA1c, postprandial lipemia, body weight, and circulating biomarkers of inflammation (8), actions that likely contribute to the benefits detected in cardiorenal outcome trials (9). Nevertheless, given the well-recognized expression of the GLP-1 receptor (GLP-1R) in the vasculature, GLP-1RAs are also believed to exert vascular actions that contribute to their cardiorenal benefits, independent of their systemic metabolic and antiinflammatory effects (10, 11).

Previous studies of the cardiorenal actions of GLP-1 medicines have identified the vascular endothelial cell (EC) as a mediator of the cardiorenal effects of GLP-1RAs. For example, liraglutide reduces blood pressure (BP) in nondiabetic mice with angiotensin II–induced (Ang II–induced) hypertension through the EC GLP-1R (12). Nevertheless, GLP-1Rs are also detected on arterial vascular smooth muscle cells (VSMCs) in multiple species, including rodents, primates, and humans (13–15). VSMC GLP-1R expression has also been localized within the kidney and in renal artery and arterioles (13, 16), which play a central role in the control of renal hemodynamics and BP.

To determine the role of the VSMC GLP-1R in mediating the BP-lowering effects of semaglutide, we generated mice with conditional inactivation of the *Glp1r* in VSMCs (*Glp1r*^VSM–/–^). These mice exhibit normal blood glucose, BP, and body weight and retain normal anorectic and glycemic responses to semaglutide. Notably, semaglutide failed to reduce BP in either normotensive or hypertensive *Glp1r*^VSM–/–^mice. The effect of semaglutide to increase glomerular filtration rate (GFR) and enhance natriuresis was also attenuated in *Glp1r*^VSM–/–^mice. In contrast, semaglutide preserved its BP-lowering actions in *Glp1r*^Tie2–/–^ mice lacking *Glp1r* in ECs and immune cells. Furthermore, semaglutide directly promoted vascular relaxation in preconstricted mesenteric arteries. Taken together, these findings identify the VSMC GLP-1R as a critical mediator for a subset of the cardiorenal actions of GLP-1RAs, linking GLP-1R signaling to BP regulation, and renal electrolyte excretion.

## Results

### Myh11-CreER^T2^ targets the VSMC Glp1r and abrogates renal and renal artery Glp1r expression.

**Figure 1:**
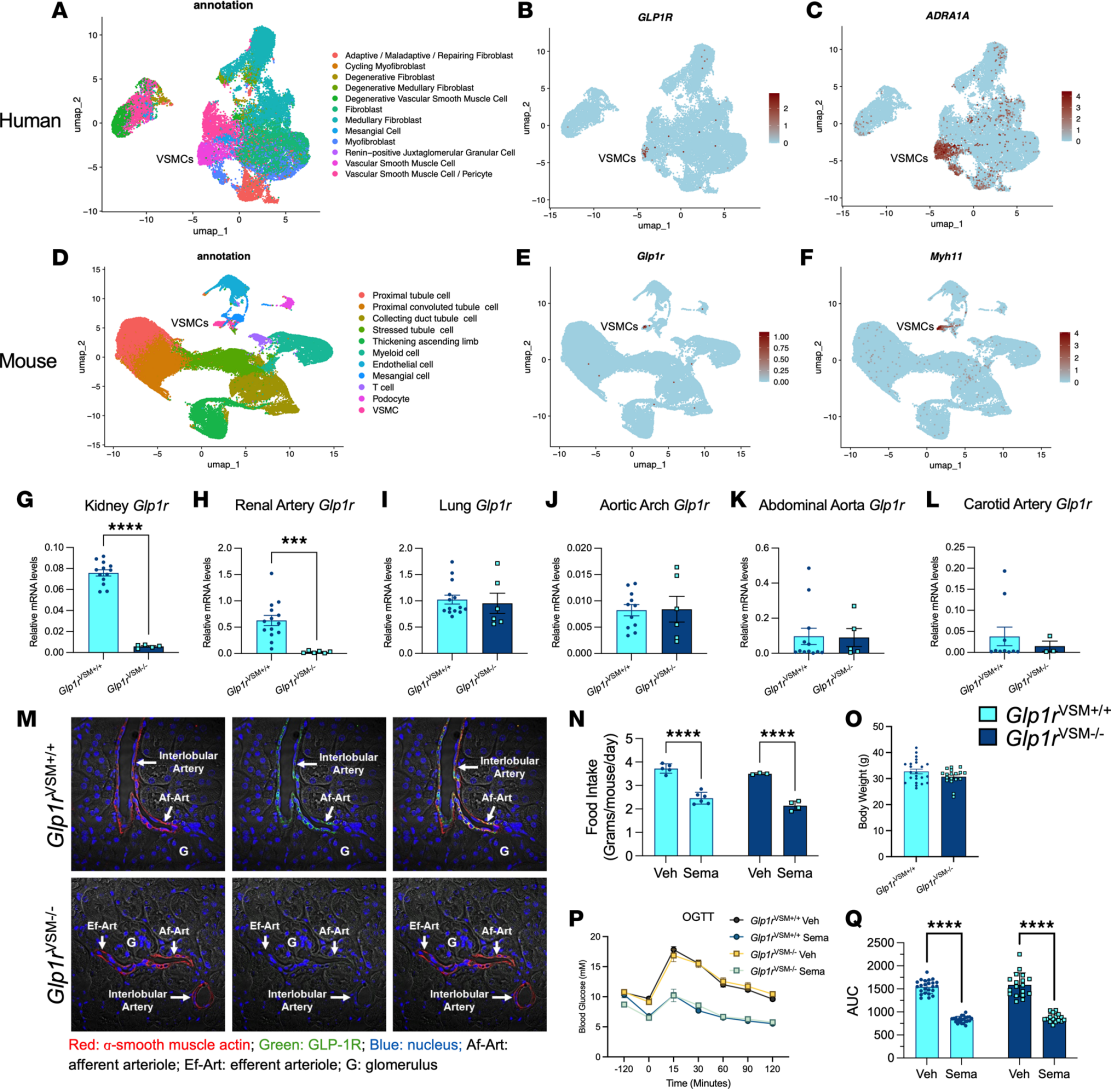
Renal GLP1R/Glp1r is expressed in human and murine vascular smooth muscle cells (VSMCs), and *Glp1r*^VSM–/–^ mice have reduced Glp1r transcripts in renal tissues. (**A**–**C**) Annotation maps of the human kidney single-cell transcriptome (18), showing expression of *GLP1R*, and vascular smooth muscle marker α1A adrenergic receptor (*ADRA1A*). (**D**–**F**) An annotation map of the mouse kidney single-cell transcriptome (17), showing expression of *Glp1r* and VSMC marker myosin heavy chain 11 (*Myh11*). (**G**–**L**) Quantification of *Glp1r* expression relative to *Rpl32* within the (**G**) kidney, (**H**) renal artery, (**I**) lung, (**J**) aortic arch, (**K**) abdominal aorta, and (**L**) carotid artery in control (*Glp1r*^VSM+/+^) and *Glp1r*^VSM–/–^ male mice (n = 5–14). (**M**) Double immunofluorescence staining of α-smooth muscle actin (left panel), GLP-1R (middle panel) and both GLP-1R and α-smooth muscle actin (right panel) in *Glp1r*^VSM+/+^ and *Glp1r*^VSM–/–^ kidney. (**N**) Average food intake per mouse within each cage of group housed mice over 24 hours with or without acute treatment with semaglutide in *Glp1r*^VSM+/+^ and *Glp1r*^VSM–/–^ mice (*n* = 4–6). (**O**–**Q**) Body weight and oral glucose tolerance with calculated area under the curve in *Glp1r*^VSM+/+^ and *Glp1r*^VSM–/–^ mice following acute semaglutide (10 μg/kg) treatment 120 minutes before oral glucose challenge (time 0) (*n* = 18–23). Data are presented as mean ± SD. ***P ≤ 0.001 and ****P ≤ 0.0001 by unpaired *t* test (**G**–**L** and **O**), or 2-way ANOVA followed by Tukey post hoc tests (**N** and **Q**). Sema, semaglutide; Veh, vehicle. Original magnification, ×31.5.

Studies using validated GLP-1R antisera have identified GLP-1R expression in VSMCs of mouse, monkey, rat, and human kidney arteries and arterioles (13, 16). To corroborate these findings prior to targeting the murine VSMC *Glp1r*, we analyzed human and murine renal transcriptomic datasets (17, 18). Kidney GLP-1R mRNA transcripts were detected primarily in VSMCs (Figure 1). In human kidney, *GLP1R* was detected in cells expressing α-1A adrenergic receptor (*ADRA1A*), a VSMC marker (19) (Figure 1, A–C). Similarly, mouse kidney single-cell transcriptomics revealed *Glp1r* expression in cells expressing smooth muscle myosin heavy chain 11 (*Myh11*), a mouse VSMC marker (20) (Figure 1, D–F).

To delete the GLP-1R in VSMCs, we used mice expressing an inducible *Myh11*-Cre recombinase to generate VSMC-specific *Glp1r*-KO mice (*Glp1r*^VSM–/–^ mice) (21). Quantitative PCR (qPCR) analysis demonstrated a 95% reduction in *Glp1r* mRNA transcript levels in the kidney and renal artery of *Glp1r*^VSM–/–^ mice (Figure 1, G and H). In contrast, *Glp1r* mRNA was not reduced in lung (which is enriched in GLP-1R–expressing ECs) (12), other major and minor blood vessels (aortic arch, abdominal aorta, aortic root, carotid artery, portal vein), cardiac chambers (atria, ventricles, interventricular septum), or brain regions such as the brain stem, hypothalamus, cortex, or cerebellum (Figure 1, I–L, and Supplemental Figure 1, A–L; supplemental material available online with this article; https://doi.org/10.1172/jci.insight.201148DS1).

Double-immunofluorescence staining of GLP-1R and α-smooth muscle actin (α-SMA) in kidney sections confirmed the absence of GLP-1R expression in VSMCs of *Glp1r*^VSM–/–^ mice (Figure 1M). In *Glp1r*^VSM+/+^ mice, GLP-1R expression was detected in VSMCs marked with α-SMA, whereas no GLP-1R signal was detected in VSMCs in *Glp1r*^VSM–/–^ mice. Analysis of GLP-1–sensitive metabolic parameters revealed that reduction of food intake in response to acute semaglutide administration was not different in *Glp1r*^VSM+/+^ versus *Glp1r*^VSM–/–^ mice (Figure 1N). Similarly, reduction of VSMC *Glp1r* expression did not perturb body weight, glucose tolerance, or the glycemic response to semaglutide (Figure 1, O–Q).

### The VSMC GLP-1R is required for semaglutide-mediated reduction in BP in normotensive and hypertensive mice.

**Figure 2:**
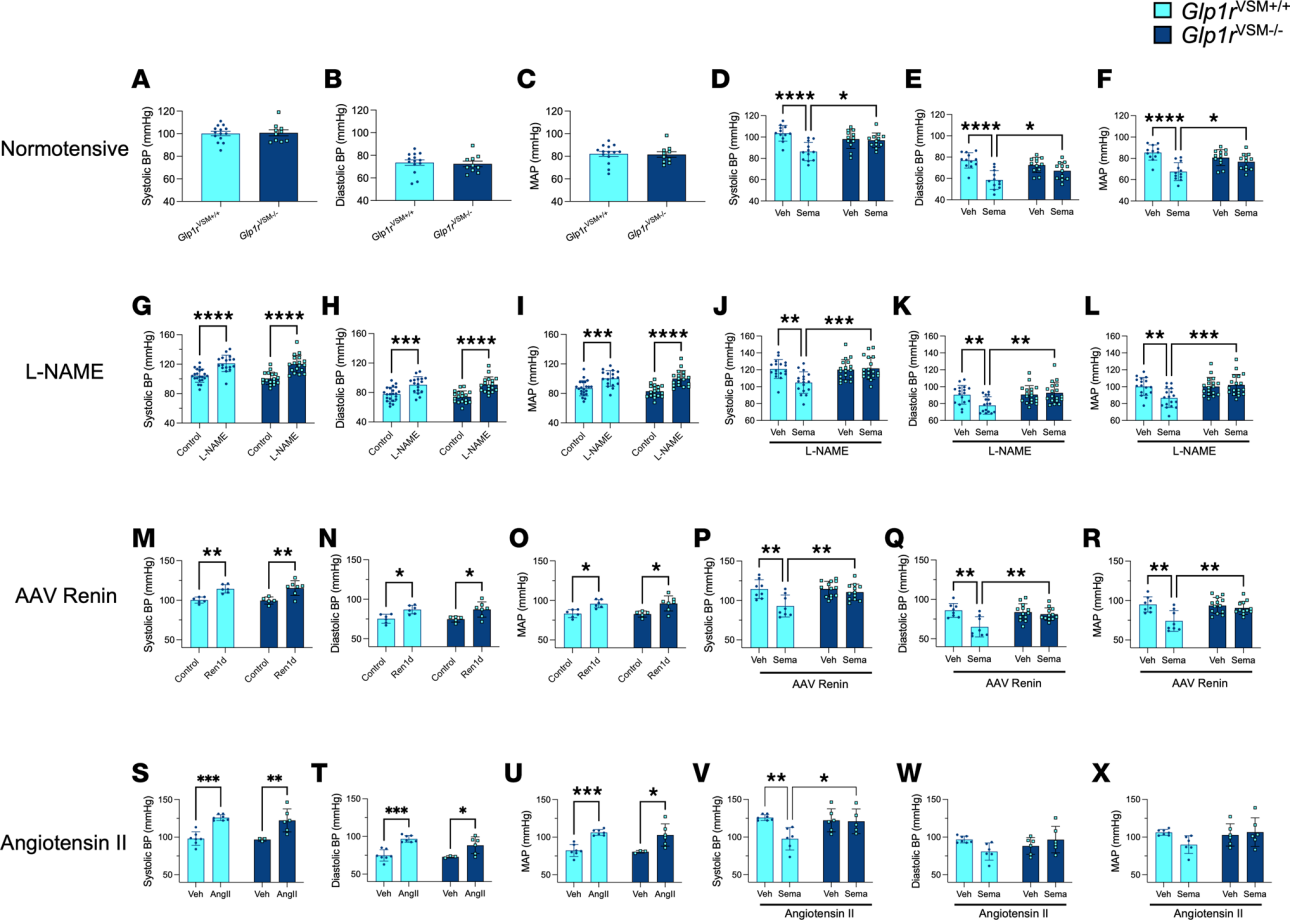
Semaglutide acutely lowers blood pressure in *Glp1r*^VSM+/+^ but not in *Glp1r*^VSM–/–^ mice. (**A**) Systolic blood pressure, (**B**) diastolic blood pressure, and (**C**) mean arterial pressure (MAP) in control *Glp1r*^VSM+/+^ and *Glp1r*^VSM–/–^ normotensive mice (*n* = 10–14). (**D**–**F**) *Glp1r*^VSM+/+^ and *Glp1r*^VSM–/–^ mice were treated acutely with semaglutide (10 μg/kg) 2 hours prior to BP measurements (*n* = 12). (**G**–**X**) BP measurements before (control) or after (**G**–**I**) induction of hypertension with 2 weeks of access to L-NAME in drinking water and (**J**–**L**) acute semaglutide treatment in L-NAME-induced hypertensive mice (*n* = 17–18), (**M**–**O**) induction of hypertension 3 weeks after AAV8-*Ren1d* viral transfection (*n* = 5–7) and (**P**–**R**) acute semaglutide treatment in AAV8-Ren1d-induced hypertensive mice (*n* = 8-13), and (**S**–**U**) induction of hypertension 3 weeks after Ang II minipump implantation (*n* = 3-7) and (**V**–**X**) acute semaglutide treatment in Ang II–induced hypertensive mice (*n* = 6-7). Data are presented as mean ± SD. *P ≤ 0.05, **P ≤ 0.01, ***P ≤ 0.001, and ****P ≤ 0.0001 by unpaired *t* test (**A**–**C**), or 2-way ANOVA followed by Tukey post hoc tests (**D**–**X**). BP, blood pressure; Sema, semaglutide; Veh, vehicle; Ang, angiotensin.

Although VSMCs and the kidney play integral roles in BP regulation, there were no baseline differences in systolic, diastolic, or mean arterial pressure (MAP) between *Glp1r*^VSM+/+^ and *Glp1r*^VSM–/–^ mice, despite a marked reduction in kidney and renal artery *Glp1r* expression in *Glp1r*^VSM–/–^ mice (Figure 2, A–C). To assess the importance of the VSMC GLP-1R for BP regulation in response to GLP-1R agonism, we treated normotensive *Glp1r*^VSM+/+^ and *Glp1r*^VSM–/–^ mice with semaglutide and recorded BP measurements. Semaglutide reduced BP in *Glp1r*^VSM+/+^ but not in *Glp1r*^VSM–/–^ mice, demonstrating that GLP-1RAs acutely lower BP through mechanisms requiring the VSMC GLP-1R (Figure 2, D–F).

We next measured the BP response to acute semaglutide using tail-cuff measurements in *Glp1r*^VSM+/+^ and *Glp1r*^VSM–/–^ mice across 3 distinct experimental models of hypertension: (a) addition of the nitric oxide synthase inhibitor L-NAME to the drinking water (Figure 2, G–L), (b) AAV8-induced renin overexpression (Figure 2, M–R), and (c) continuous Ang II infusion with s.c. osmotic minipumps (Figure 2, S–X). *Glp1r*^VSM+/+^ and *Glp1r*^VSM–/–^ mice achieved similar levels of hypertension under each of the 3 modalities, indicating that loss of the VSMC GLP-1R does not exacerbate the severity of experimental hypertension. Moreover, a single injection of semaglutide lowered BP in hypertensive *Glp1r*^VSM+/+^ but not *Glp1r*^VSM–/–^ mice, demonstrating that the BP-lowering response to semaglutide is preserved in a hypertensive setting (Figure 2, J–L, P–R, and V–X). The tail cuff BP measurements were replicated using radiotelemetry implants in freely moving mice with Ang II–induced hypertension treated acutely with the GLP-1RA liraglutide, further implicating the VSMC GLP-1R as essential for the BP-lowering effects of GLP-1 medicines (Supplemental Figure 2).

To determine whether sustained semaglutide administration lowers BP in these models, we treated hypertensive (L-NAME in drinking water) *Glp1r*^VSM+/+^ and *Glp1r*^VSM–/–^ mice with daily semaglutide for 3 weeks. Repeated semaglutide continued to reduce BP in hypertensive *Glp1r*^VSM+/+^ but not in *Glp1r*^VSM–/–^ mice (Supplemental Figure 3, A–C). Consistent with these findings, left ventricular mass and heart weight were lower in hypertensive semaglutide-treated *Glp1r*^VSM+/+^ mice but not in *Glp1r*^VSM–/–^ mice (Supplemental Figure 3, D and E).

Intriguingly, levels of *Glp1r* mRNA transcripts were increased in kidney but not the renal artery after 2 weeks of daily semaglutide treatment (Supplemental Figure 4, A and B). To determine if *Glp1r* mRNA levels differ between the renal artery and arterioles, we microdissected the renal vascular tree. *Glp1r* expression was higher in afferent arterioles than in the renal artery (Supplemental Figure 4C).

Since hypertension can alter signaling responses across the vascular tree (conduit artery versus arterioles) (22), we examined whether *Glp1r* mRNA expression changes over the course of hypertension. After 4 weeks of hypertension induced by AAV8 renin overexpression or continuous Ang II infusion, kidney and renal artery *Glp1r* expression was unchanged (Supplemental Figure 4, D and E). In contrast, after 30 weeks of AAV8 renin-induced hypertension, kidney *Glp1r* mRNA levels were increased, indicating a time-dependent upregulation during prolonged hypertensive stress (Supplemental Figure 4D).

We next examined the expression of genes associated with vascular remodeling. In the kidney and renal artery of *Glp1r*^VSM+/+^ and *Glp1r*^VSM–/–^ mice, mRNA levels of the pro-α1 chains of type 1 and 3 collagen (*Col1a1* and *Col3a1*), abundant forms of collagen in the vascular wall (23), were unchanged (Supplemental Figure 4, F, G, I, and J). However, *Col5a1* mRNA (corresponding to *COL5A1*, encoding a semaglutide-regulated collagen protein in humans; ref. 24) was increased in both tissues in normotensive *Glp1r*^VSM–/–^ mice (Supplemental Figure 4, H and K). The selective increase of *Col5a1* in *Glp1r*^VSM–/–^ mice suggests that loss of the VSMC GLP-1R may subtly alter vascular extracellular matrix composition.

### GLP-1R expression within the Tie2 domain is dispensable for the BP-lowering effects of semaglutide.

**Figure 3:**
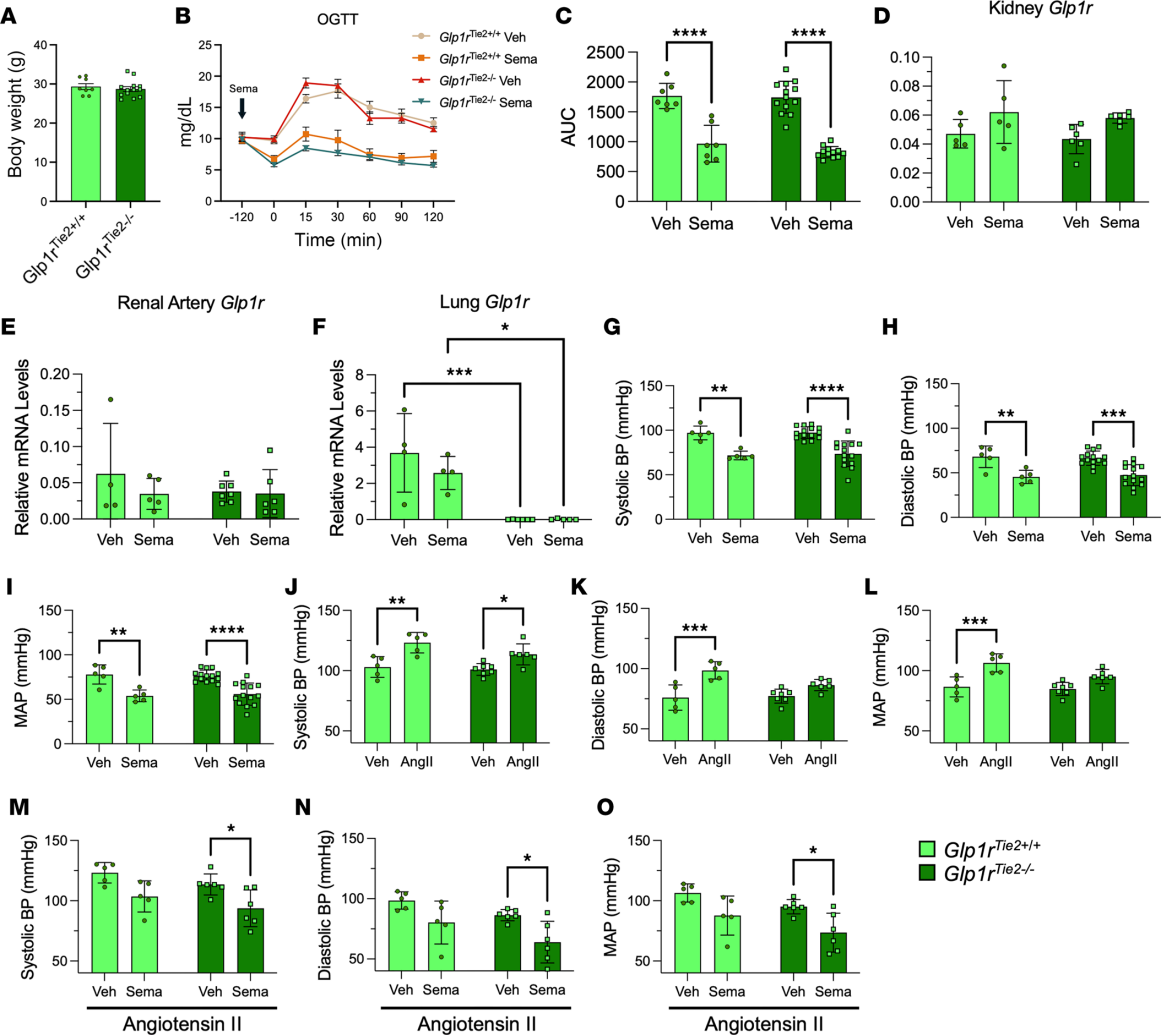
Loss of *Glp1r* expression in the Tie2 domain does not impair semaglutide-mediated reductions in BP. (**A**–**C**) Body weight and oral glucose tolerance test with calculated area under the glucose curve in control *Glp1r*^Tie2+/+^ and *Glp1r*^Tie2–/–^ male mice with acute semaglutide (10 μg/kg) treatment 120 minutes before glucose challenge (*n* = 7–13). (**D**–**F**) Quantification of gene expression relative to *Rpl32* within the kidney, renal artery, and lung in *Glp1r*^Tie2+/+^ and *Glp1r*^Tie2–/–^ male mice (*n* = 4–6). (**G**–**I**) Systolic blood pressure, diastolic blood pressure, and mean arterial pressure (MAP) in control and *Glp1r*^Tie2–/–^ male mice treated acutely with semaglutide 120 minutes prior to BP measurements (*n* = 5-14). (**J**–**O**) BP measurements following (**J**–**L**) induction of hypertension with Ang II for 2 weeks and (**M**–**O**) acute semaglutide treatment in angiotensin II-induced hypertensive mice (*n* = 5–7). Data are presented as mean ± SD. *P ≤ 0.05, **P ≤ 0.01, ***P ≤ 0.001, and ****P ≤ 0.0001 by unpaired *t* test (**A**), or 2-way ANOVA followed by Tukey post hoc tests (**C**–**O**). BP, blood pressure; Sema, semaglutide; Veh, vehicle; Ang, angiotensin.

Previous studies of *Cdh5*-Cre GLP-1R–KO mice with inactivation of the EC *Glp1r* suggested that EC GLP-1Rs are required for the BP-lowering effect of liraglutide in mice with Ang II–induced hypertension (12). To reconcile these observations, we studied *Glp1r*^Tie2–/–^ mice with reduction of *Glp1r* expression in ECs and hematopoietic cells within the Tie2^+^ expression domain (25, 26). Body weight, glucose tolerance, and the glycemic response to semaglutide were similar in *Glp1r*^Tie2+/+^ and *Glp1r*^Tie2–/–^ male (Figure 3, A–C) and female mice (Supplemental Figure 5, A–C). Levels of *Glp1r* mRNA were not different in the kidney or renal artery of *Glp1r*^Tie2+/+^ versus *Glp1r*^Tie2–/–^ mice but were markedly reduced in the lung of *Glp1r*^Tie2–/–^ mice, a tissue enriched in ECs (Figure 3, D–F). Hence, hematopoietic and EC Tie2^+^ lineages do not contribute significantly to kidney *Glp1r* expression. Notably, semaglutide reduced BP in normotensive *Glp1r*^Tie2+/+^ and *Glp1r*^Tie2–/–^ mice (Figure 3, G–I, and Supplemental Figure 5, D–F), indicating that GLP-1R expression in Tie2^+^ cells is not required for semaglutide-mediated reduction of BP in healthy mice.

We next evaluated BP responses in *Glp1r*^Tie2–/–^ mice with hypertension induced by continuous Ang II infusion. Two weeks after hypertension induction, systolic BP increased to a similar extent in *Glp1r*^Tie2+/+^ and *Glp1r*^Tie2–/–^ mice; however, the rise in diastolic BP was blunted in Ang II–infused *Glp1r*^Tie2–/–^ mice (Figure 3, J–L). Importantly, acute semaglutide treatment reduced BP (systolic, diastolic, and MAP) in both *Glp1r*^Tie2+/+^ and *Glp1r*^Tie2–/–^ mice (Figure 3, M–O). Collectively, our results demonstrate that mice lacking hematopoietic and EC *Glp1r* expression in the Tie2^+^ domain retain BP-lowering responses to semaglutide under both normotensive and hypertensive conditions.

### Semaglutide increases diuresis and natriuresis through VSMC GLP-1R–dependent and –independent mechanisms.

**Figure 4:**
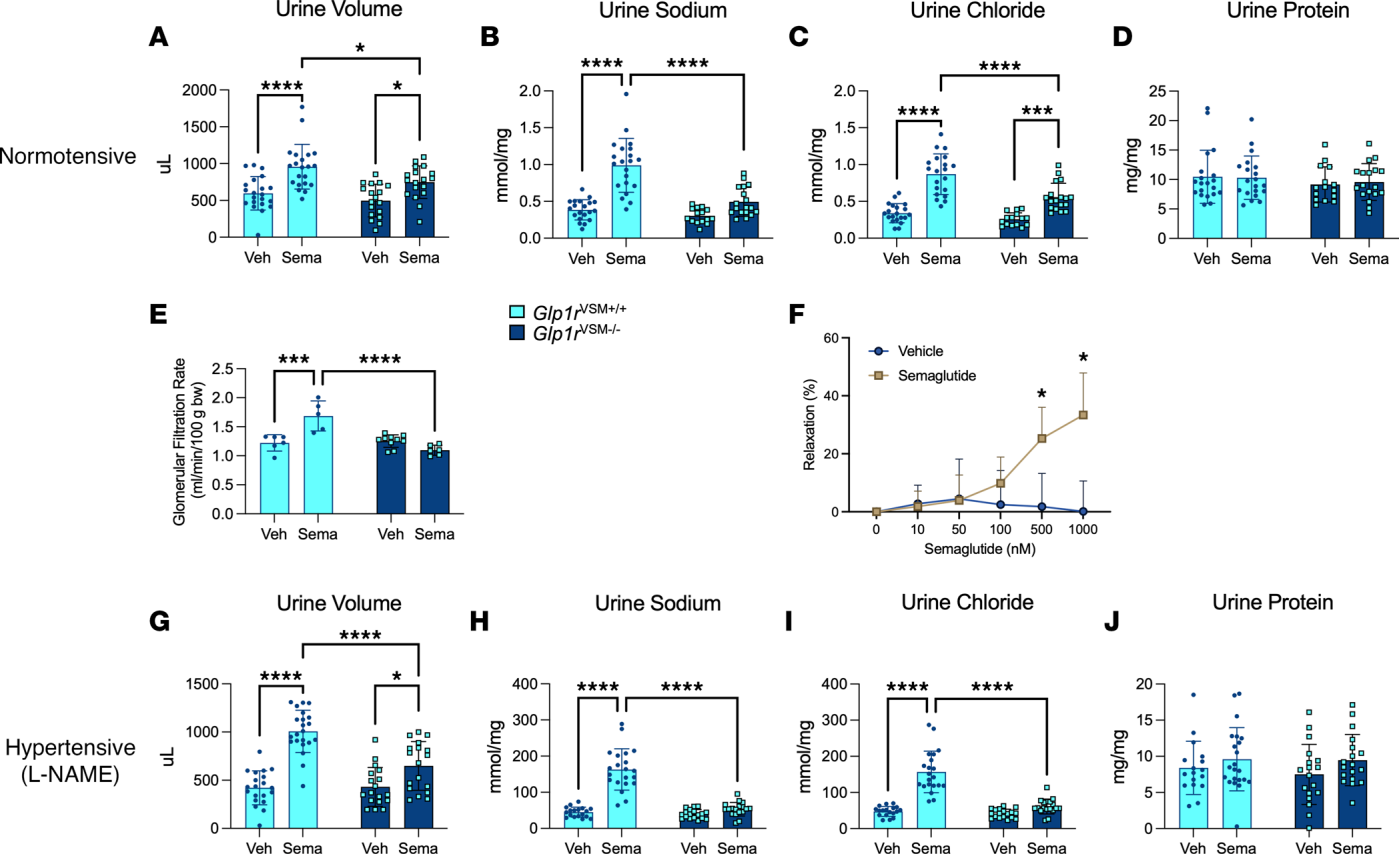
Semaglutide-induced diuresis and natriuresis are lower but not ablated by loss of the VSMC GLP-1R. (**A**–**D**) Urine volume, urine sodium, urine chloride, and urine protein in normotensive control *Glp1r*^VSM+/+^ and *Glp1r*^VSM–/–^ male mice treated acutely with semaglutide (10 μg/kg) 60 minutes prior to water gavage; urine was collected 180 minutes following gavage (*n* = 15–22). (**E**) Glomerular filtration rate was measured in *Glp1r*^VSM+/+^ and *Glp1r*^VSM–/–^ mice treated acutely with semaglutide (10 μg/kg) 30 minutes before a bolus of FITC- sinistrin (*n* = 5–10). (**F**) Myography readings depicting vasorelaxation of ex vivo mesenteric arteries, assessed after phenylephrine preconstriction (1 μM), in response to semaglutide (10–1,000 nM). (**G**–**J**) Two weeks after L-NAME–induced hypertension, mice were treated acutely with semaglutide (i.p.) 60 minutes prior to a water gavage, and urine was collected 180 minutes later. (**G**–**J**) Urine volume, urine sodium, urine chloride, and urine protein were measured (*n* = 18–22). Urine sodium, chloride, and protein levels were normalized to creatinine concentration. Data are presented as mean ± SD. *P ≤ 0.05, ***P ≤ 0.001, and ****P ≤ 0.0001 by 2-way ANOVA followed by Tukey post hoc-tests (**A**–**E** and **G**–**J**) or multiple unpaired *t* tests (**F**). Sema, semaglutide; Veh vehicle.

One proposed mechanism by which GLP-1RAs lower BP is through changes in blood volume via their promotion of diuresis (increased urine output) and natriuresis (enhanced urinary sodium excretion). These effects reduce circulating volume and vascular filling pressure, which in turn can contribute to reductions in systemic BP (27–29). To evaluate whether loss of GLP-1RA–stimulated renal water and solute excretion could explain the lack of BP reduction in response to semaglutide in *Glp1r*^VSM–/–^ mice, we treated mice with a water gavage and collected urine 3 hours thereafter. Semaglutide increased urine volume in both *Glp1r*^VSM+/+^ and *Glp1r*^VSM–/–^ mice; however, this effect was relatively greater in *Glp1r*^VSM+/+^ mice (Figure 4A). Thus, the magnitude of semaglutide-induced diuresis is lower, but not completely ablated, with loss of the VSMC GLP-1R. Similarly, semaglutide increased natriuresis in *Glp1r*^VSM+/+^ mice, as shown by higher urine sodium and chloride concentrations (normalized to urine creatinine levels)—actions attenuated, but not completely eliminated, in *Glp1r*^VSM–/–^ mice (Figure 4, B and C). These differences do not seem to be secondary to global changes in renal permeability, as urinary protein concentrations (normalized to urine creatinine levels) were unchanged (Figure 4D).

To determine the role of the VSMC GLP-1R in GLP-1RA–induced renal hemodynamic responses, GFR was measured in *Glp1r*^VSM+/+^ and *Glp1r*^VSM–/–^ mice following acute semaglutide injection. Semaglutide increased GFR in *Glp1r*^VSM+/+^ mice but not in *Glp1r*^VSM–/–^ mice (Figure 4E). We next examined whether semaglutide could directly modulate vasorelaxation in WT arteries ex vivo. In isolated murine mesenteric arteries preconstricted with phenylephrine, semaglutide promoted relaxation in a dose-dependent manner (Figure 4F).

To further assess whether renal excretory effects reflect the BP response, we examined the acute effects of GLP-1R activation on renal excretion in L-NAME–hypertensive mice. Semaglutide significantly increased diuresis and natriuresis in *Glp1r*^VSM+/+^ mice, whereas the diuretic effect was blunted and natriuresis absent in *Glp1r*^VSM–/–^ mice (Figure 4, G–I). Urine protein excretion was unchanged in both *Glp1r*^VSM+/+^ and *Glp1r*^VSM–/–^ mice (Figure 4J). These results indicate that semaglutide-induced renal excretory responses are only partially dependent on the VSMC GLP-1R and can be dissociated, at least in part, from BP reduction.

### Loss of the VSMC GLP-1R affects basal and semaglutide-regulated renal artery and kidney proteome responses to semaglutide.

**Figure 5:**
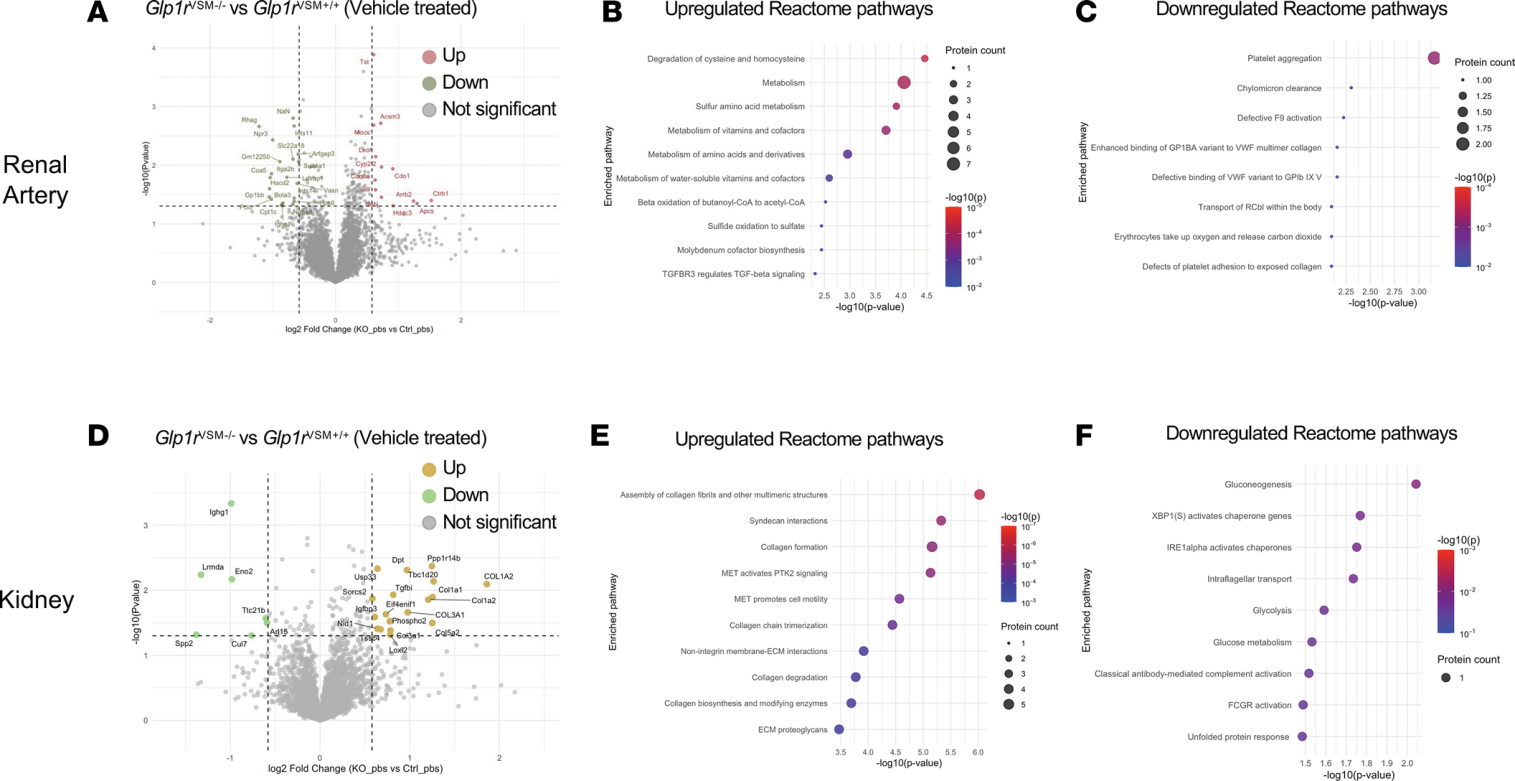
Identification of renal artery and kidney proteomic changes in *Glp1r*^VSM+/+^ versus *Glp1r*^VSM–/–^ mice. (**A**) Volcano plot depicting changes in the distribution of significance and fold change of identified proteins in renal artery between *Glp1r*^VSM+/+^ and *Glp1r*^VSM–/–^ mice. (**B** and **C**) Feature plots for identified Reactome pathways (**B**) positively and (**C**) negatively correlated with VSMC *Glp1r* knockdown. The color scheme is based on p-value distribution. (**D**) Volcano plot depicting changes in quantified protein between *Glp1r*^VSM+/+^ and *Glp1r*^VSM–/–^ mice in the kidney. (**E** and **F**) Feature plots for identified Reactome pathways (**E**) positively and (**F**) negatively correlated with VSMC Glp1r KO. Statistical comparisons were made using unpaired *t* test between groups.

We next examined differences in the tissue proteome between *Glp1r*^VSM+/+^ and *Glp1r*^VSM–/–^ mice under basal conditions in saline-treated mice. In the renal artery, we detected clear differences between genotypes, with 13 proteins upregulated and 20 proteins downregulated in *Glp1r*^VSM+/+^ versus *Glp1r*^VSM–/–^ (Figure 5A), despite no detectable basal BP differences in these mice (Figure 2). Pathway analysis of the upregulated proteins in *Glp1r*^VSM–/–^ renal arteries revealed enrichment in processes related to cysteine and homocysteine degradation, sulfur amino acid metabolism, vitamin metabolism, and general amino acid metabolism (Figure 5B). In contrast, downregulated proteins were mainly associated with pathways involving platelet aggregation, chylomicron clearance, and defective coagulation factor IX activation, among others (Figure 5C). Similarly, we identified 19 upregulated proteins in *Glp1r*^VSM–/–^ kidneys, primarily linked to collagen formation and extracellular matrix organization, along with 7 downregulated proteins associated with gluconeogenesis, XBP1 activity, and other metabolic functions (Figure 5, D–F). Although the smaller number of differentially expressed proteins in the kidney compared with the renal artery limits broader conclusions, these findings indicate that chronic loss of VSMC GLP-1R signaling is accompanied by detectable basal proteomic alterations at both the vascular and organ level.

We next compared renal artery proteomes from semaglutide- and saline-treated *Glp1r*^VSM+/+^ and *Glp1r*^VSM–/–^ mice. Male mice were treated with semaglutide or vehicle, and 4 hours later, the renal artery and kidney were obtained for analysis of protein content.

**Figure 6:**
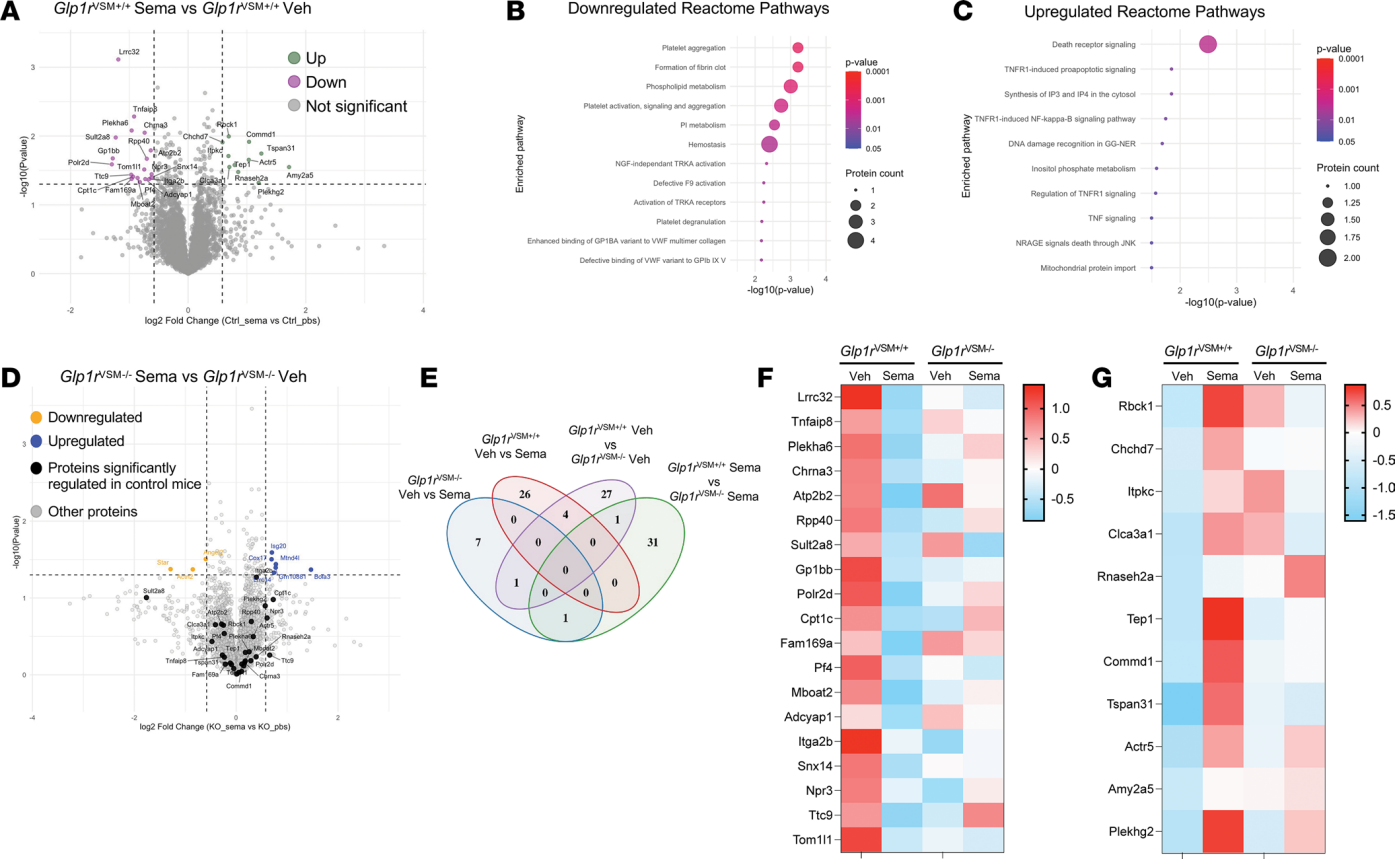
Identification of renal artery proteomic changes with semaglutide treatment in *Glp1r*^VSM+/+^ and *Glp1r*^VSM–/–^ mice. (**A**) Mice were treated with semaglutide (10 μg/kg) 4 hours before renal artery excision and processing. (**A**) Volcano plot depicting changes in the distribution of significance and fold change of identified proteins between control mice treated with vehicle (Veh) and semaglutide (Sema). (**B** and **C**) Feature plots for identified Reactome pathways (**B**) positively and (**C**) negatively correlated with vehicle versus semaglutide treatment. The color scheme is based on *P* value distribution. (**D**) Volcano plot depicting changes in quantified protein between *Glp1r*^VSM–/–^ mice treated with vehicle or semaglutide; highlighted proteins represent significantly changed targets in control mice. (**E**) Venn diagram depicting overlap of proteins regulated within the separate comparisons. (**F** and **G**) Heatmaps comparing proteins in control and *Glp1r*^VSM–/–^ mice that are (**F**) positively and (**G**) negatively correlated with semaglutide treatment. Statistical comparisons were made using unpaired *t* test between groups.

In renal arteries from *Glp1r*^VSM+/+^ mice, semaglutide administration was associated with downregulation of 19 proteins and upregulation of 11 proteins compared with saline-treated controls (Figure 6A). Pathway enrichment analysis revealed that the downregulated proteins were primarily related to platelet aggregation, fibrin clot formation, and phospholipid metabolism, whereas upregulated proteins mapped to death receptor signaling and proapoptotic pathways (Figure 6, B and C). These proteomic signatures may reflect adaptive vascular responses accompanying acute semaglutide-induced reductions in BP and enhanced renal function. In contrast, renal arteries from *Glp1r*^VSM–/–^ mice exhibited only minor proteomic alterations after semaglutide treatment (9 proteins in total), none of which overlapped with the changes observed in *Glp1r*^VSM+/+^ mice (Figure 6D). Notably, the proteomic signatures identified in the renal artery of *Glp1r*^VSM+/+^ mice were completely absent in *Glp1r*^VSM–/–^ mice, consistent with the loss of semaglutide-induced hemodynamic and renal effects in these animals (Figure 6, D–G).

**Figure 7:**
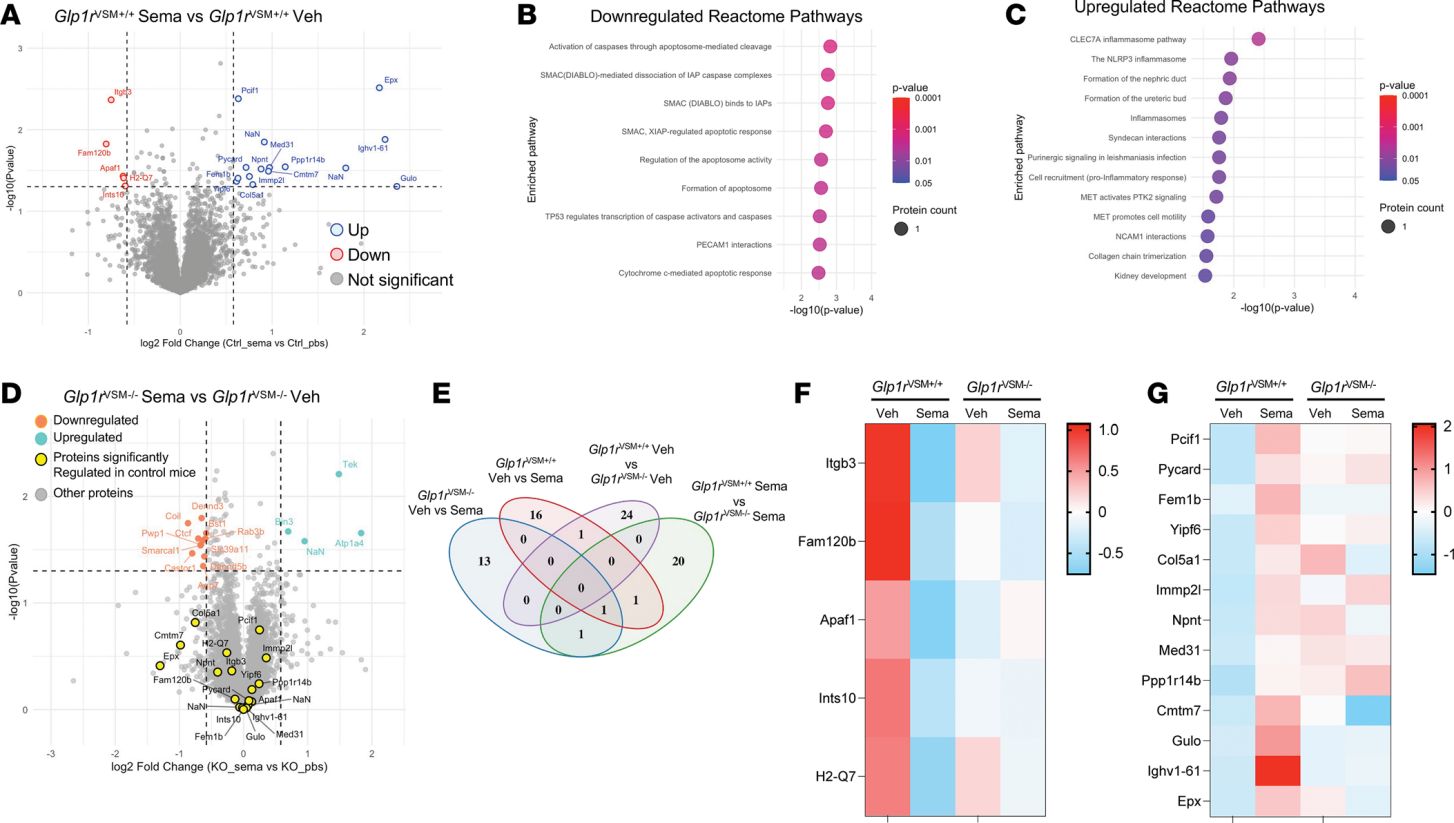
Identification of kidney proteomic changes with semaglutide treatment in *Glp1r*^VSM+/+^ and *Glp1r*^VSM–/–^ mice. Mice were treated with semaglutide (10 mg/kg) 4 hours before kidney excision and processing. (**A**) Volcano plot depicting changes in the distribution of significance and fold change of identified proteins between control mice treated with vehicle (Veh) and semaglutide (Sema). (**B** and **C**) Feature plots for identified Reactome pathways (**B**) positively and (**C**) negatively correlated with vehicle versus semaglutide treatment. The color scheme is based on *P* value distribution. (**D**) Volcano plot depicting changes in quantified protein between *Glp1r*^VSM–/–^ mice treated with vehicle or semaglutide; highlighted proteins represent significantly changed targets in control mice. (**E**) Venn diagram depicting overlap of proteins regulated within the separate comparisons. (**F** and **G**) Heatmaps comparing proteins in control and *Glp1r*^VSM–/–^ mice that are (**F**) positively and (**G**) negatively correlated with semaglutide treatment. Statistical comparisons were made using unpaired *t* test between groups.

Global proteomic analysis was also performed on whole-kidney tissue from saline- and semaglutide-treated mice (Figure 7). In *Glp1r*^VSM+/+^ mice, semaglutide induced modest but detectable proteomic shifts, with downregulation of 5 proteins enriched in pathways linked to apoptotic signaling, including caspase activation, second mitochondria-derived activator of caspases–dependent pathways and general regulators of programmed cell death. In parallel, 13 proteins were upregulated, mapping to inflammatory and developmental pathways, including CLEC7a (Dectin-1) signaling, NLRP3 inflammasome activation, and processes involved in nephric duct and ureteric bud formation (Figure 7, A–C). These observations may reflect secondary tissue remodeling and/or paracrine signaling responses initiated by semaglutide. In contrast, these semaglutide-responsive proteins were not regulated in *Glp1r*^VSM–/–^ kidneys (Figure 7, D–G). Although a small number of proteins were altered in *Glp1r*^VSM–/–^ mice following semaglutide administration (Figure 7, D and E), they did not overlap with those observed in *Glp1r*^VSM+/+^ mice. Together, these data suggest that the proteomic changes detected in the renal artery and kidney following systemic semaglutide administration are dependent on the VSMC GLP-1R.

## Discussion

GLP-1 medicines induce diuresis and natriuresis and reduce BP as well as albuminuria, an established marker of kidney dysfunction (30, 31). However, deciphering the renal mechanisms of GLP-1 action has been challenging, complicated in part by low levels of renal GLP-1R expression and prior misattribution of GLP-1R localization in the kidney (32). Our interrogation of human and mouse kidney scRNA-seq datasets highlights heterogeneity in VSMC expression of *GLP1R/Glp1r*. Though GLP-1R^+^ cells are a relatively small subset of VSMCs, *Glp1r*^VSM–/–^ mice with Cre expression driven by the *Myh11* promoter exhibit complete loss of immunofluorescence staining of GLP-1R in kidney sections and 95% knockdown of *Glp1r* mRNA expression in both renal artery and kidney (Figure 1). Thus, VSMCs are the major GLP-1R–expressing cell type in mouse renal tissue.

GLP-1 medicines lower BP in animals and humans, a potential contributing factor to the lowered risk of MACE detected in cardiovascular outcome trials (2). The pathways that transduce GLP-1RA–mediated reduction of BP are not well defined, and kidney-centric mechanisms such as inhibition of tubular salt reabsorption, vascular inflammatory immune cell infiltration, central regulation of vascular tone, and either EC- or VSMC-regulated vasodilation have been proposed (33). Here, we show that the acute weight loss–independent actions of semaglutide to lower BP are ablated in *Glp1r*^VSM–/–^ mice, identifying an essential role for the VSMC GLP-1R as the BP-lowering target for semaglutide. Notably, semaglutide reduced BP in experimental models of hypertension induced by L-NAME, AAV-mediated-renin overexpression, and Ang II infusion. These effects occurred independently of changes in glycemic control or body weight in nonobese, nondiabetic *Glp1r*^VSM+/+^ mice.

In hypertensive control animals (induced by L-NAME), chronic semaglutide administration for 2–5 weeks reduced BP, left ventricular mass, and total heart weight, whereas these cardioprotective effects were absent in *Glp1r*^VSM–/–^ mice. Taken together, our data support the contention that the VSMC GLP-1R likely contributes to a subset of the cardio-renal benefits of GLP-1 medicines. Furthermore, we show an increase in kidney *Glp1r* expression following repeated, daily treatment of semaglutide. If these observations are replicated in humans, they may contribute to our understanding of the efficacy of GLP-1 medicines in reducing chronic hypertension and protecting the kidney.

Somewhat surprisingly, in contrast to previous studies with liraglutide (12), *Glp1r*^Tie2–/–^ mice display intact BP lowering responses to semaglutide. Moreover, *Glp1r*^Tie2–/–^ mice do not display differences in renal artery or kidney *Glp1r* expression, as opposed to *Glp1r*^VSM–/–^ mice in which *Glp1r* expression is markedly reduced in these structures, further highlighting that VSMCs are the main renal GLP-1R–expressing cell type. However, a few methodological differences may contribute to the incongruency of our findings with those of Helmstädter et al. (12). Though the same dose of Ang II was used, the s.c. minipumps had different durations and release rates. Our pump duration of 4 weeks (versus 1 week) allows more time for the maintenance of hypertension, extending recovery time after surgical implantation, and allowing for stabilization of hypertension, which can take up to 2 weeks to reach a plateau (34). Moreover, *Glp1r*^Cdh5–/–^ mice studied previously as a model for KO of the EC *Glp1r* displayed much higher systolic BP (~155 mmHg) (12) than current findings in our *Glp1r*^Tie2–/–^ mice (~125 mmHg), revealing phenotypic differences in our mouse models and limiting direct comparability of our findings.

One hypothesis for the BP-lowering actions of GLP-1 medicines invokes reduction in blood volume secondary to enhanced renal water excretion (27). Although semaglutide increased diuresis and natriuresis, these effects were attenuated but not completely ablated by loss of the VSMC GLP-1R. In contrast, changes in BP in response to semaglutide were absent in *Glp1r*^VSM–/–^ mice. Thus semaglutide-induced renal excretion of water and sodium does not regulate BP by inducing hypovolemia. Rather, our data raise the possibility that VSMCs in preglomerular renal vasculature transduce changes in hemodynamics in response to GLP-1RAs, which contribute to lower BP in mice. Specifically, GFR was increased in semaglutide-treated *Glp1r*^VSM+/+^ but not in *Glp1r*^VSM–/–^ mice, without changes in urine protein content, suggesting that this finding is independent of renal barrier integrity. Furthermore, we show that semaglutide promotes relaxation of the mesenteric artery ex vivo, supporting direct hemodynamic actions of GLP-1 medicines in peripheral vasculature.

Proteomic profiling of the renal artery, where GLP-1R expression is most pronounced in the kidney, revealed that semaglutide remodels pathways related to platelet aggregation, fibrin clot formation, lipid metabolism, and proapoptotic signaling in *Glp1r*^VSM+/+^ mice. These signatures are consistent with adaptive vascular responses that may contribute to reduced vascular resistance, improved perfusion, and ultimately enhanced sodium and water excretion. In striking contrast, these semaglutide-induced proteomic changes were completely absent in *Glp1r*^VSM–/–^ mice, mirroring the loss of hemodynamic responses in this setting. The lack of overlap between *Glp1r*^VSM+/+^ mice and *Glp1r*^VSM–/–^ proteomic responses strongly support a causal role for the VSMC GLP-1R in renal artery remodeling following GLP-1RA treatment.

Whole-kidney proteomics provided complementary insights. In *Glp1r*^VSM+/+^ mice, semaglutide induced modest but detectable changes, including downregulation of apoptotic regulators and upregulation of proteins involved in inflammatory and developmental pathways. Although these signatures were distinct from those observed in the renal artery, they may reflect cross-talk between vascular and parenchymal compartments. The absence of similar proteomic changes in *Glp1r*^VSM–/–^ kidneys reinforces the concept that VSMC GLP-1R signaling links semaglutide action to renal adaptations. Notably, although whole-kidney proteomic remodeling was modest, several proteins regulated by semaglutide in our mouse studies, including COL5A1, have also been identified as semaglutide-responsive targets in humans. Circulating proteomic analyses from the STEP 1 and STEP 2 clinical trials revealed reductions in COL5A1 levels following semaglutide treatment (24). Furthermore, the expression of *Col5a1* was increased in *Glp1r*^VSM–/–^ kidney and renal artery compared with *Glp1r*^VSM+/+^ tissue (Supplemental Figure 4, H and K). This translational overlap underscores the relevance of our findings and provides independent support for the validity of the observed proteomic signatures.

Despite the development of new renoprotective medicines (35), men and women living with T2D continue to experience high rates of chronic kidney disease (36), often progressing to a requirement for supportive care and, ultimately, renal replacement therapy, including dialysis and kidney transplantation (35). Findings that GLP-1 medicines such as semaglutide are renoprotective in people with T2D and obesity (6, 7) provide new options for improving renal outcomes and decreasing the likelihood of developing end stage kidney disease. Identification of the mechanisms linking GLP-1R signaling to control of BP and the biology of renal artery and kidney protein expression extends our understanding of GLP-1 action and highlights the importance of the VSMC as a key GLP-1R^+^ cell type contributing to the cardiorenal actions of GLP-1 medicines such as semaglutide.

### Limitations.

The *Myh11* promoter is extensively used as a smooth muscle cell Cre driver (20) and is regarded as the most cell type–restrictive and specific promoter available to study VSMCs. In the *Myh11-CreER^T2^* mouse (and, thus, the *Glp1r*^VSM–/–^ mouse), the transgene was inserted on the Y chromosome precluding the study of female mice. Furthermore, the majority of studies presented herein is fairly acute and carried out in mice without obesity or diabetes, limiting generalization of the findings to these important metabolic comorbidities. Whether the VSMC is similarly important for the BP-lowering and renal actions of GLP-1 medicines in humans will require further interrogation.

## Methods

### Sex as a biological variable.

Wherever possible, both male and female mice were tested to ensure the validity of key responses in both sexes. In the *Glp1r*^VSM–/–^ mouse, the transgene is inserted on the Y chromosome, precluding the study of female mice in this model.

### Animal models and experiments.

Mice were housed up to 5 per cage at The Centre for Phenogenomics (TCP) specific pathogen–free mouse facility (Mount Sinai Hospital, Toronto, ON, Canada) on a 12-hour light/dark cycle at 23°C with ad libitum access to standard rodent chow diet (18% kcal from fat, 2018 Harlan Teklad) and acidified drinking water. Deletion of *Glp1r* in VSMCs of mice with C57BL/6 background (*Glp1r*^VSM–/–^) were generated by crossing tamoxifen-inducible *Myh11-*Cre*ER^T2^* mice (21) (Jackson Laboratory, 019079) with *Glp1r*^fl/fl^ mice (37, 38), provided by R. Seeley, University of Michigan, Ann Arbor, Michigan, USA. Only male *Glp1r*^VSM–/–^ mice were used due to Y chromosome expression of the *Myh11-CreER^T2^* transgene. Conditional Cre-induced inactivation of the *Glp1r* gene was carried out via 5 consecutive daily gavages of 100 μL of 10 mg/mL tamoxifen (Sigma-Aldrich T5648) diluted in corn oil (1 mg/mouse) after mice reached 8 weeks of age. Control animals were pooled from *Glp1r*^fl/fl^ and *Myh11*-Cre*ER^T2^*–positive mice, all treated with tamoxifen. These control genotypes did not differ in any of the key parameters of this study, including body weight, fasting blood glucose, glucose tolerance, semaglutide-induced blood glucose lowering, BP, urinary markers, kidney and renal artery *Glp1r* gene expression, and induction of hypertension (data not shown). *Glp1r*^Tie2–/–^ mice were generated by crossing *Glp1r*^fl/fl^ mice with Tg (Tek-Cre)1Ywa (*Tie2)* Cre mice (Jackson Laboratory, 008863) to target endothelial and immune cells in the Tie2 domain, as previously described (25, 26). Control animals consisted of pooled mice from Tie2-Cre^+^ mice and *Glp1r*^fl/fl^ littermates. Animals were acutely treated by i.p. injection, or repeatedly: once daily by s.c. injection with 10 μg/kg of semaglutide (Sema) (Novo Nordisk) or phosphate-buffered saline (Vehicle [Veh]).

### Double-immunofluorescence staining.

Double-immunofluorescence staining of GLP-1R and α-SMA in kidney sections was performed as previously described (39, 40). Briefly, 4 μm paraffin sections were incubated overnight at 4°C with a GLP1R antibody (ab218532; rabbit monoclonal IgG, 1:200; Abcam, Cambridge, UK) and an α-SMA antibody (14-9760-82; mouse monoclonal IgG, 1:200; Invitrogen, Massachusetts, USA). Sections were then incubated for 1 hour at room temperature with fluorescent secondary antibodies (ab150080, Alexa Fluor 594 goat anti–rabbit IgG, 1:1,000; and ab150113, Alexa Fluor 488 goat anti–mouse IgG, 1:1,000; Abcam). All slices were set in an antifade mounting medium with DAPI (H-1500; Vector Laboratories, CA, USA). All images were captured with a Zeiss LSM 700 laser scanning microscope.

### Analysis of kidney scRNA-seq data.

Published mouse kidney (GSE160048) (17) and human kidney (GSE183277) (18) scRNA-seq datasets were retrieved and analyzed with a standard pipeline to sctransform, PCA calculation, UMAP projection, and clustering using Seurat 5 in R (41). The human dataset consisted of healthy (45 donors) and diseased kidneys (48 patients) while the mouse data analyzed a timeline of acute kidney injury for up to 14 days after unilateral ischemia/reperfusion-induced injury.

### Oral glucose tolerance tests.

For oral glucose tolerance testing, mice were fasted 5 hours and treated with semaglutide (i.p.; 10 μg/kg) or vehicle 2 hours prior to glucose. Oral glucose (Sigma) was administered by gavage at 1.5 g/kg (0.15 g/mL glucose in water). Glucose was measured (Contour glucometer) in tail vein blood taken at time 0, 15, 30, 60, 90, and 120 minutes after glucose gavage.

### Food intake.

Mice were given preweighed food in home cages for 24 hours following an acute dose of semaglutide (i.p. 10 μg/kg) or vehicle. Values were averaged per mouse in the group-housed cages.

### Measurement of BP.

Noninvasive BP recordings were performed using a tail cuff Coda Monitor System (Kent Scientific), which has been validated against telemetry monitoring by our group and others (42). BP was recorded after a minimum of 2 days acclimation at approximately 10:00 a.m. to adapt mice to the procedure and to minimize stress. Acute semaglutide treatments were performed on 2 separate days with a 1-day washout period in between, during which PBS was administered before recording. Within each treatment day, the average of at least 5 independent BP measurements was used for final data points, and consecutive PBS treatment days were averaged together.

To implant BP telemeters, mice were anesthetized initially with 5% isoflurane in an oxygen stream and maintained on 2%–3% isoflurane. Mice were kept on a heating pad (38°C) throughout implantation with a BP telemeter (43). All mice were allowed a minimum period of 1 week to recover from device implantation surgery prior to initiation of data collection. Systolic BP was measured in conscious, freely moving mice using an implanted radiotelemetry device (PA-C10, Data Sciences International, St. Paul, MN). Measurement was recorded for 840 minutes following liraglutide administration.

### Induction of hypertension.

Hypertension was induced in mice for a minimum of 2 weeks prior to BP measurements or before initiation of daily semaglutide treatment. L-NAME drinking water–induced hypertension was achieved by acclimating mice with water in water bottles before adding L-NAME (Sigma, N5751) at a concentration of 1 mg/mL to the drinking water (44). Adeno-Associated Virus (AAV) Renin-induced hypertension was achieved via tail vein injection of AAV8-TBG-m-*Ren1d* (F61R/P65S) (3 × 10^10^ genome copies) (45) purchased from Vector Biolabs. The nucleotide sequence encoding mouse *Ren1d* was placed downstream of the liver-specific thyroxine-binding globulin (TBG) promoter and inserted into AAV8 vector to target the liver (46). The mouse *Ren1d* (F61R/P65S) mutation was created enabling a cleavage site from prorenin in nonrenal tissues. In this way, the liver renin overexpression does not directly impact renal biology (47), allowing the study of kidney response to changes in BP. ANG II–induced hypertension was achieved by s.c. implantation of Alzet osmotic pumps (Model 1004 Alzet) filled with Ang II (~0.6mg/kg/day; Sigma, A9525) (12). Osmotic pumps filled with PBS were used as controls.

### Myography.

Vasorelaxation of mesenteric arteries was assessed by wire myography after phenylephrine (PE) preconstriction (1 μM) in response to semaglutide (10–1000 nmol/L). Male C57BL/6 mice (10 weeks old) were anesthetized with isoflurane, and mesenteric arteries were then isolated and placed in ice-cold Krebs–Henseleit solution, containing 112 mM NaCl, 5 mM KCl, 25 mM NaHCO_3_, 1 mM NaH_2_PO_4_, 0.5 mM MgCl_2_, 2.5 mM CaCl_2_, and 11.5 mM glucose. Then, mesenteric arteries were cut into rings (2 mm in length) and further mounted onto the wire myograph system before being allowed to warm up to 37°C for 10 minutes. Resting tension of the arteries was set according to manufacturer’s protocol. Mesenteric artery vasoreactivity was measured using a high-potassium solution (KPSS, Krebs-Hanseleit solution with 80 mM KCl), followed by a wash with Krebs-Henseleit solution. Vessels were allowed to equilibrate for 30 minutes. Vasorelaxation of mesenteric arteries was examined by precontraction with PE (1 μM) before treatment with increasing concentrations of Semaglutide (10–1,000 nmol/L).

### Echocardiography.

A Vevo 2100 Ultrasound Biomicroscope was used to achieve noninvasive, high-resolution ultrasound imaging of cardiac structure in mice. Echocardiography ultrasound (30–40 MHz; Vevo770, VisualSonics) was carried out after 4 weeks of access to L-NAME drinking water and 2 weeks of daily semaglutide treatment. Left ventricular mass was assessed in live mice anesthetized with isoflurane (2%–3%), with body temperature maintained on a warming platform and continuously monitored via rectal thermometer. The operator was blinded to the genotype and treatment of mice for ultrasound measurements and subsequent analysis.

### Urine collection.

Semaglutide was injected i.p. (10 μg/kg); 1 hour later, mice were gavaged with water (20 μL per gram body weight) and singly placed in dry, sterile, empty cages with a wire bottom without bedding. Mice were allowed to urinate for 3 hours in these cages before returning to the home cage. Urine was collected from the wire bottom and cage floor with a pipette and cell scraper and stored in a 2 mL sterile tube and stored at –80°C.

### Blood and tissue collection.

Mice were sacrificed by CO_2_ inhalation, blood was collected by cardiac puncture, and tissues were dissected, weighed, and immediately frozen in liquid nitrogen. Tibia length was measured using digital calipers. For isolation of cardiac subregions, hearts were rinsed in PBS and a cannula passed through the aorta into the left ventricle. Methylene blue dye (1% w/v in PBS) was injected into the left ventricle and incubated for 5 minutes. Then, the heart was cut on the short axis, rinsed in PBS, and frozen in Optimal Cutting Temperature medium. A dissecting microscope was used to scrape samples from cryosection slides for subsequent RNA analysis. All blood samples were collected from cardiac puncture and mixed with a 10% volume of TED (5,000 kIU/mL Trasylol, MilliporeSigma, A6279; 32 mM EDTA; and 0.01 mM Diprotin A, MilliporeSigma, I-9759). Blood samples were kept on ice, and plasma was collected shortly afterward by centrifugation (5,000*g*) for 5 minutes and stored at −80°C.

### Urine analyte measurements.

Urine analytes were measured using the BioRad Liquid Assayed MultiQual for creatinine, sodium, chloride, and total protein. Samples were analyzed by the Pathology Phenogenomics Core at TCP, Mount Sinai Hospital, Toronto, ON.

### Microdissection.

Male C57BL/6 mice (10 weeks old) were anesthetized with isoflurane. Renal arteries and lungs were harvested for qPCR. Kidneys were removed and sliced along the corticomedullary axis. Slices were placed in ice-cold glucose DMEM. Afferent arterioles were microdissected under a stereomicroscope (SMZ1500; Nikon, Yuko, Japan).

### Tissue gene expression analysis.

For the extraction of mRNA, tissue samples were homogenized in TRI Reagent (Molecular Research Center) using a TissueLyser II system (Qiagen) and then chloroform extracted, precipitated using isopropanol, washed with 75% ethanol, and reconstituted with DEPC-treated water. First-strand cDNA was synthesized from DNase I–treated total RNA using SuperScript III and random hexamers (Thermo Fisher Scientific). Reverse transcription reactions were performed for 10 minutes at 25°C, 50 minutes at 50°C, and an additional 15 minutes at 70°C. Gene expression levels were quantified by qPCR using a QuantStudio 5 System and TaqMan Gene Expression Master Mix and Assays (Thermo Fisher Scientific). Gene expression levels of *Glp1r* (Mm00445292_m1), *Col1a1* (Mm00801666_g1), *Col3a1* (Mm01254476_m1), and *Col5a1* (Mm00489299_m1) were calculated as 2^–ΔCT^ relative to the reference gene *Rpl32* (Mm02528467_g1). Microdissected renal arteries, lung, and isolated afferent arterioles were transferred to RLT buffer (RNeasy Mini Kit; Qiagen, Venlo, Netherlands) for RNA extraction. The time for dissection was < 30 minutes after euthanasia to avoid RNA degradation. The extracted RNA samples were treated with DNase I (AM2222; Thermo Fisher Scientific, Waltham, MA) to avoid the contamination of genomic DNA. Total RNA was reverse transcribed into cDNA with a reverse transcription system using oligo(dT) primer.

### GFR measurement.

GFR in conscious mice was determined via transcutaneously measured elimination kinetics of fluorescein isothiocyanate– sinistrin (FITC-sinistrin) (39). A transdermal GFR monitor (Medibeacon) placed on a depilated area of the skin of the mouse was used to record fluorescence intensity. Mice were lightly anesthetized with isoflurane and received an injection of a single bolus of FITC-sinistrin solution (7 mg/100 g body weight) via the retroorbital venous sinus. The fluorescence signal was collected through the transdermal GFR monitor for 2 hours, and the GFR was calculated from the measured FITC-sinistrin clearance half-life using Medibeacon software.

### Proteomics.

Kidney and renal artery proteomic analysis was performed on samples from *Glp1r*^VSM+/+^ and *Glp1r*^VSM–/–^ mice treated with semaglutide (i.p. 10 μg/kg) or vehicle for 4 hours before tissue collection. A minimum of 6 biological replicates was analyzed per group. For proteomic analysis, both renal arteries or half of one kidney was lysed in 5% SDS, 50 mM triethylammonium bicarbonate using a TissueLyser II system (Qiagen). Protein concentration was measured using the Pierce BCA Protein Assay. A total of 25 μg of protein was processed per sample. Samples were reduced with dithiothreitol at 10 mM for 30 minutes at 56°C and alkylated with iodoacetamide at 30 mM for 45 minutes at room temperature in the dark. Each sample was brought up to a final volume of 50 μL. MagReSyn HILIC beads (6.25 μL per sample) were transferred to a microcentrifuge tube and washed 2 times with 200 μL Equilibration buffer (15% ACN in 100 mM Ammonium acetate, pH 4.5). Each 6.25 μL initial bead slurry was transferred to a new microcentrifuge tube in equilibration buffer. In total, 50 μL Binding buffer (30% ACN in 200 mM Ammonium acetate, pH 4.5) was added to 50 μL of protein solution for a final volume of 100 μL. Equilibration buffer was removed from the beads and 100 μL protein solution was added and mixed at 1200 rpm for 30 minutes at room temperature. Beads were washed twice with 200 μL Wash buffer (95% ACN) and mixed for 1 minute at 1,200 rpm. All wash buffer was removed, and beads were resuspended in 100 μL digestion buffer (50 mM Tris-HCl, pH 8.0) containing 1 μg trypsin. Protein was digested for 1 hour at 47°C with mixing (1,200 rpm). Peptides were collected and acidified to 2% formic acid. Peptides were desiccated and stored at –80°C until mass spectrometry (MS) acquisition.

For data-independent acquisition (DIA) liquid chromatography–tandem MS (LC-MS/MS), 500 ng protein equivalent of digested peptides were analyzed using a nano-high-performance liquid chromatography coupled to MS. The sample was loaded onto Evotip Pure per manufacturer instructions. Peptides were eluted from the column (catalog EV-1137, 15 cm × 150 μm with 1.5 μm beads) with the 30 samples per day method preformed acetonitrile gradient generated by an Evosep One system and analyzed on a timsTOF Pro 2. The column toaster was set to 40°C. The total DIA protocol was 44 minutes. The MS1 scan had a mass range of 100–1,700 Da in dia-PASEF. TIMS settings were accumulation and ramp time of 100 ms, and within the mobility range (1/K0) of 0.6 to 1.6 V·s/cm^2^, with a cycle time of 2 seconds. For MS2, 1 mobility window with 2 ramps was used for 32 mass windows, 29.8 Da wide with 5 Da mass overlap. The mobility range was from 0.61/K0 to 1.451/K0. This was at a duty cycle of 100% and a ramp rate of 9.52Hz. In total, 1+ ions were excluded from fragmentation using a polygonal filter. The auto calibration was off. Spectronaut v20 directDIA+ workflow was used to search the data with the Spectronaut generated mouse spectral library (Mouse_PDB_2023). Parameters for the search were default. Differential abundance testing used was unpaired 2-tailed *t* test.

### Statistics.

Data are represented as the mean ± SD. Statistical comparisons were made, where appropriate, by Student’s *t* test, or 2-way ANOVA followed by Tukey post hoc test using GraphPad Prism version 10 software. Values considered outliers using Grubbs’ test were excluded from analysis. *P* ≤ 0.05 was considered statistically significant.

### Study approval.

All animal experiments were approved by the Animal Care and Use Subcommittee at the TCP at Mount Sinai Hospital (Toronto, Canada).

### Data availability.

Values for all data points in graphs are reported in the Supporting Data Values file. Proteomics data has been deposited as a complete submission to the MassIVE repository (https://massive.ucsd.edu/ProteoSAFe/static/massive.jsp) and assigned the accession no. MSV000100883. The ProteomeXchange accession no. is PXD074593.

## Author contributions

KDM, JAK, LLB, XC, YC, JF, EL, MGK, VR, SK, and JZ executed mouse experiments. KDM, JAK, LLB, MJGR, CKW, JZ, VR, SK, and JW performed data analysis. DJD, LLB, and KDM designed the experiments. DJD and KDM wrote the manuscript. All authors reviewed and edited the manuscript prior to submission.

## Conflict of interest

DJD has received consulting fees from Amgen, Alnylam, AstraZeneca Inc., Crinetics, Eli Lilly, General Medicines Inc., Kallyope, Metsera, Pfizer Inc., Protagonist Therapeutics Inc., and Sanofi and speaking fees from Novo Nordisk Inc. within the past 12 months. Mt. Sinai Hospital has received investigator-initiated grant support from Amgen, Eli Lilly Inc., and Zealand Pharmaceuticals Inc. to support preclinical studies in the Drucker lab.

## Funding support

- Banting and Best Diabetes Centre Novo Nordisk Chair in Incretin Biology to DJD
- Sinai Health-Novo Nordisk Foundation Chair in Regulatory Peptides to DJD
- CIHR grants 154321 and 192044 to DJD
- Diabetes Canada-Canadian Cancer Society Grant OG-3- 24-5819-DD to DJD
- National Institutes of Health grant R01HL168098 to JW
- American Heart Association Transformational Project Award 24TPA1294438 to JW
- National Institutes of Health grant R01DK134616 to JZ
- Canadian Institutes of Health Research Fellowship Award MFE – 200948 to KDM
- Banting & Best Diabetes Centre post-doctoral fellowship to KDM
- Kidney Research Scientist Core Education and National Training (KRESCENT) postdoctoral fellowship (cosponsored by the Kidney Foundation of Canada, the Canadian Society of Nephrology, and Canadian Institutes of Health Research) to KDM
- European Union’s Horizon Europe research and innovation programme under the Marie Skłodowska-Curie grant agreement No 101105823 to MJGR

## Supplementary Material

Supplemental data

Supporting data values

## Version 1 03/03/2026

In-Press Preview

## Version 2 04/22/2026

Electronic publication
