# Supplementary material for: Semaglutide reduces murine blood pressure through the vascular smooth muscle GLP-1 receptor
Source: JCI Insight. 2026 Mar 3;11(8):e201148. doi: 10.1172/jci.insight.201148 (PMC13135390; doi:10.1172/jci.insight.201148)
Supplement: Supplemental data [file jciinsight-11-201148-s163.pdf]

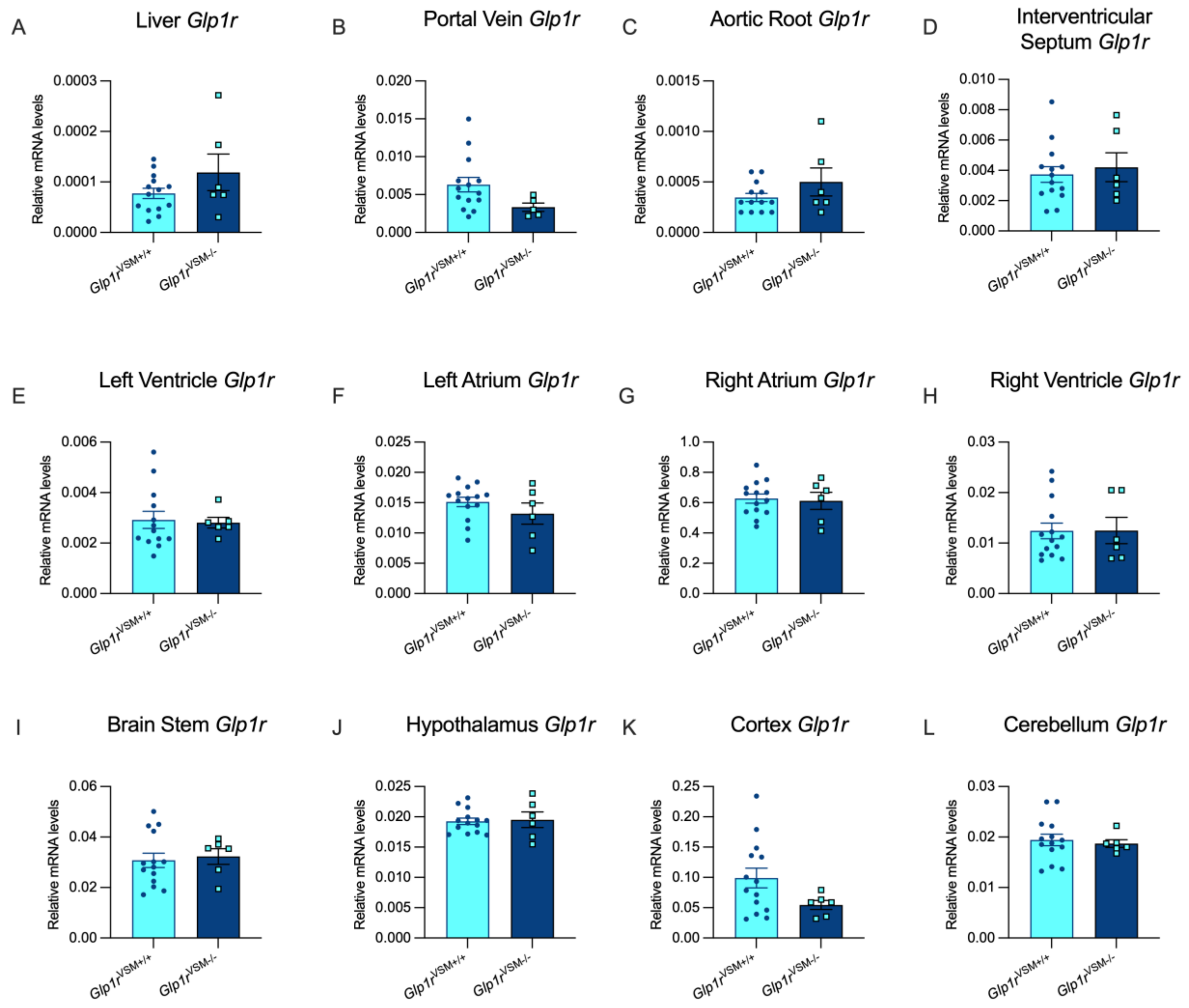


Supplemental Figure 1

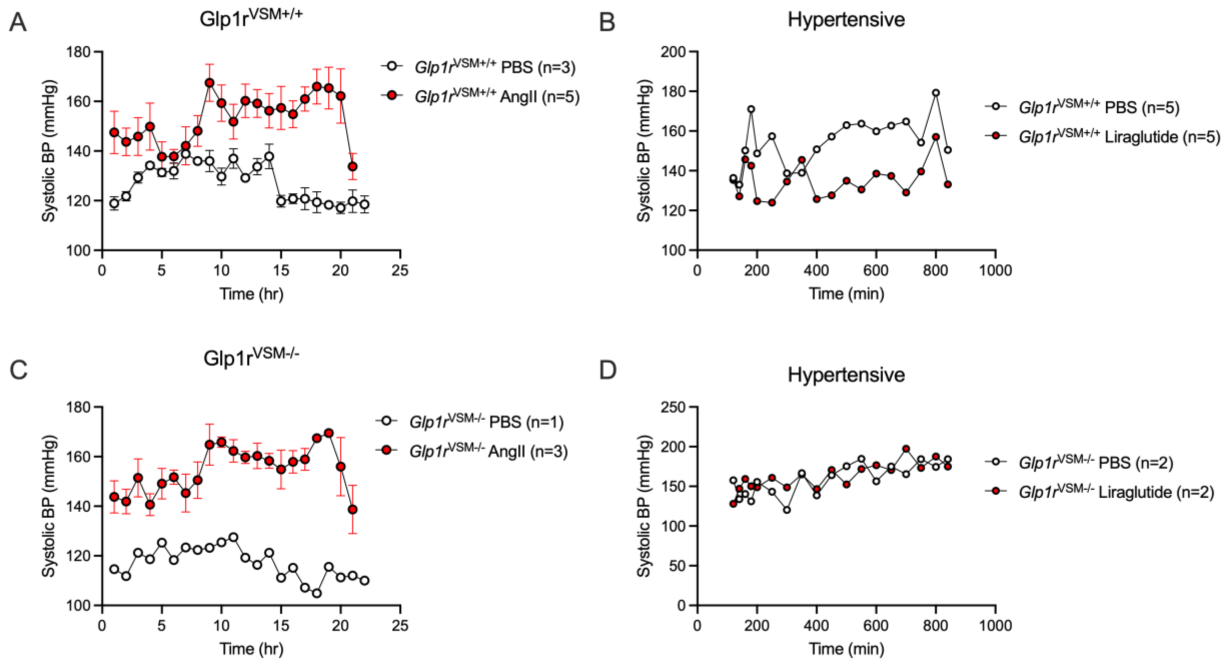


Supplemental Figure 2

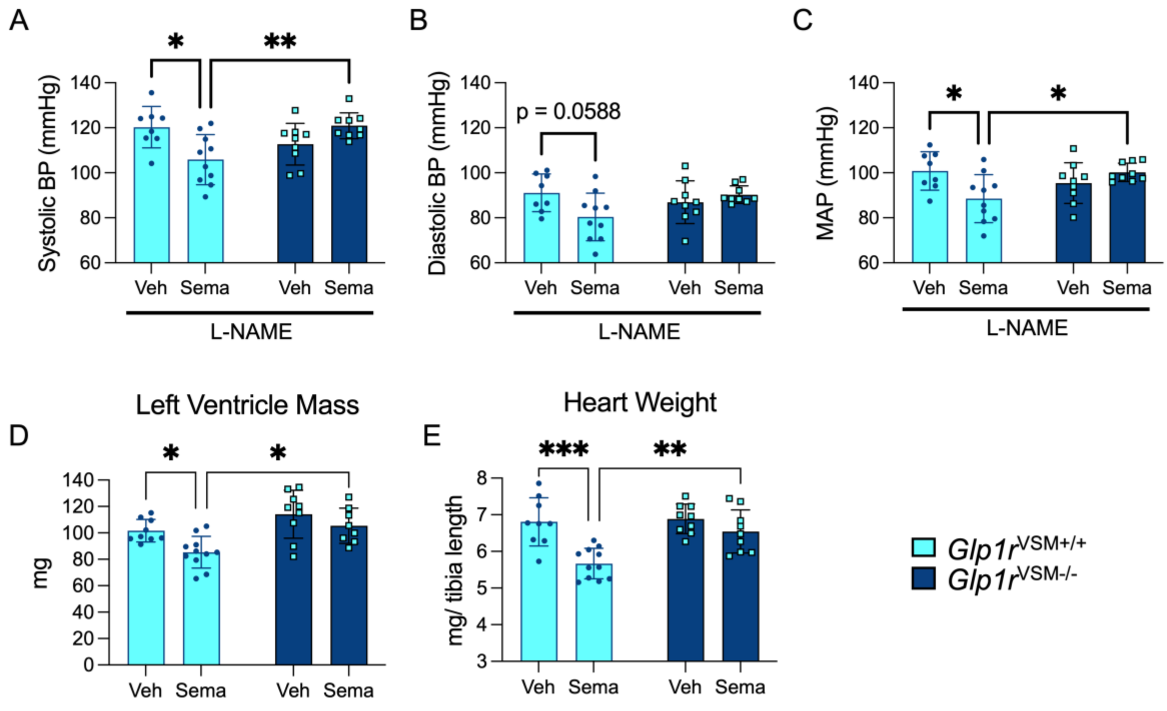


Supplemental Figure 3

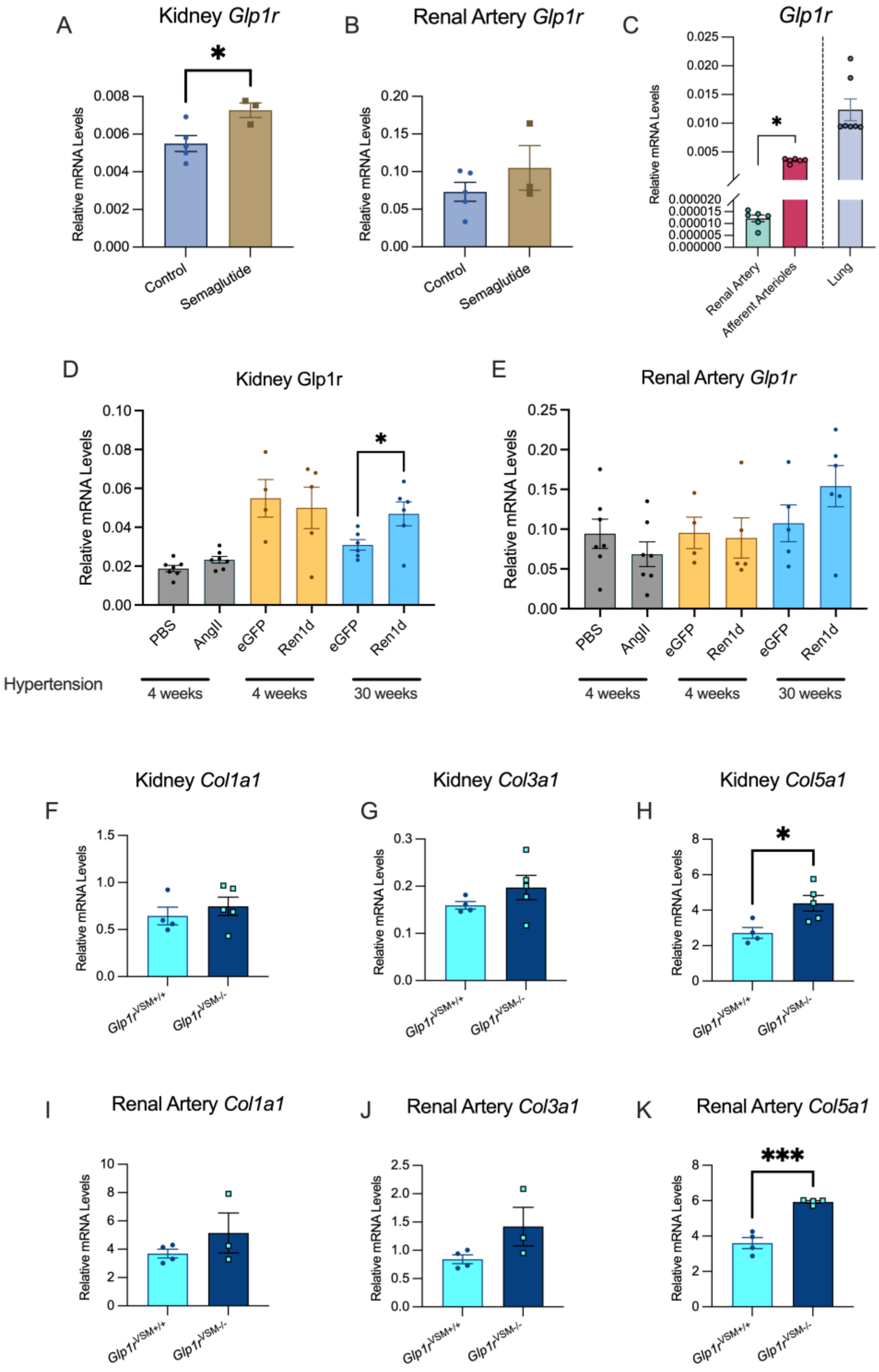


Supplemental Figure 4

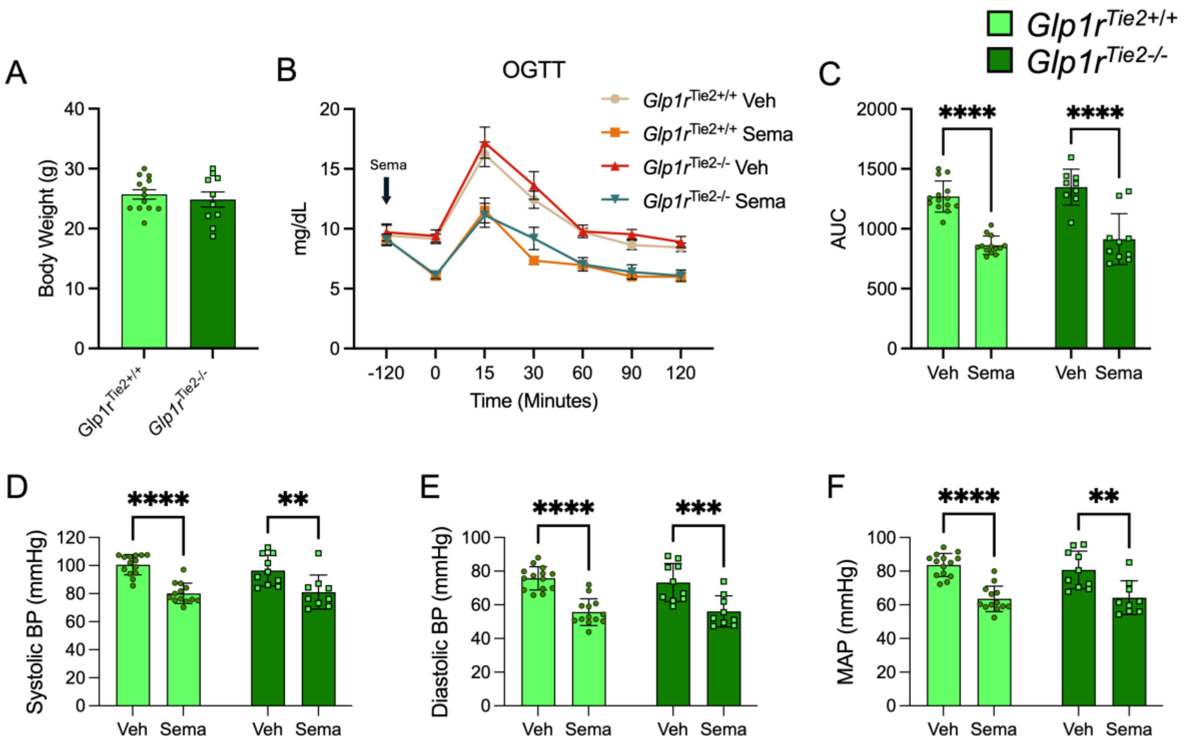


Supplemental Figure 5

**Supplemental Figure 1: *Glp1r* gene expression is similar in non-renal tissues of control and *Glp1r*$^{VSM-/-}$ mice.**
Quantification of *Glp1r* gene expression relative to *Rpl32* within the (A) liver, (B) portal vein, (C) aortic root, (D) interventricular septum, (E) left ventricle, (F) left atrium, (G) right atrium, (H) right ventricle, (I) brain stem, (J) hypothalamus, (K) cerebral cortex, and (L) cerebellum in control (*Glp1r*$^{VSM+/+}$) and *Glp1r*$^{VSM-/-}$ male mice (n = 6–13). Data are presented as mean ± SD and statistically compared by student's t-test.

**Supplemental Figure 2: Liraglutide acutely lowers blood pressure in *Glp1r*$^{VSM+/+}$ but not in *Glp1r*$^{VSM-/-}$ mice.**
Systolic BP measured by radio telemetry in control *Glp1r*$^{VSM+/+}$ and *Glp1r*$^{VSM-/-}$ mice. (A) induction of hypertension in *Glp1r*$^{VSM+/+}$ mice after angiotensin 2 minipump implantation (n=3-5) and (B) acute liraglutide treatment in Ang II-induced hypertensive *Glp1r*$^{VSM+/+}$ mice (n=5). (C) induction of hypertension in *Glp1r*$^{VSM-/-}$ mice after Ang II minipump implantation (n=1-3) and (D) acute liraglutide treatment in Ang II -induced hypertensive *Glp1r*$^{VSM-/-}$ mice (n=2).

**Supplemental Figure 3: Semaglutide lowers blood pressure in hypertensive *Glp1r*$^{VSM+/+}$ but not *Glp1r*$^{VSM-/-}$ mice after chronic semaglutide treatment.**
(A-C) *Glp1r*$^{VSM+/+}$ and *Glp1r*$^{VSM-/-}$ male mice were provided L-NAME in the drinking water for 2 weeks to induce hypertension before beginning daily semaglutide treatment. After three weeks of daily semaglutide (10 ug/kg) treatment (A) systolic blood pressure, (B) diastolic blood pressure, and (C) mean arterial pressure (MAP) were measured in hypertensive mice following an acute dose of semaglutide (10 ug/kg) administered 120 minutes prior to BP measurements (n=8-11). (D-E) After two weeks of daily semaglutide treatment in hypertensive mice (D) left ventricle mass was measured by echocardiography (n=8-11). After five weeks of semaglutide treatment in hypertensive mice (E) total heart weight was measured at termination and normalized to tibia length (n=8-11). Data are presented as mean ± SD. *P ≤ 0.05, **P ≤ 0.01, and ***P ≤ 0.001 by 2-way ANOVA followed by Tukey post-hoc tests. BP, blood pressure; Sema, semaglutide; Veh, vehicle.

**Supplemental Figure 4: Comparison of changes in *Glp1r* and collagen gene expression in renal tissues of *Glp1r*$^{VSM+/+}$ and *Glp1r*$^{VSM-/-}$ mice.**
(A-B) Comparison of *Glp1r* gene expression in normotensive *Glp1r*$^{VSM+/+}$ mice following two weeks of daily semaglutide (5ug/kg; IP) treatment. (C) Comparison of *Glp1r* gene expression between microdissected renal artery, microdissected kidney afferent arterioles, and whole lung tissue (n=6-7). Comparison of *Glp1r* gene expression in (D) kidney and (E) renal artery between control and hypertensive animals for a duration of 4 or 30 weeks (n=4-7). Quantification of Collagen alpha1 chain 1,3, and 5 gene expression relative to *Rpl32* within the (F-H) kidney and (I-K) renal artery (n=3-5). Data are presented as mean ± SD and statistically compared by student's t-test. *P ≤ 0.05, and ***P ≤ 0.001.

**Supplemental Figure 5: Loss of GLP-1R expression in the Tie2 domain does not impact**

**semaglutide-mediated reduction of blood pressure in female mice.**

(A) Body weight and (B) oral glucose tolerance with (C) calculated area under the glucose curve in female control *Glp1r*$^{Tie2+/+}$ and *Glp1r*$^{Tie2-/-}$ female mice with acute semaglutide treatment (10 μg/kg) 120 minutes before glucose challenge (n=10-14). (D) Systolic blood pressure, (E) diastolic blood pressure, and (F) mean arterial pressure in control and *Glp1r*$^{Tie2-/-}$ mice treated acutely with semaglutide 120 minutes prior to measurement (n=9-14). Data are presented as mean ± SD. **P ≤ 0.01, ***P ≤ 0.001, and ****P ≤ 0.0001 by 2-way ANOVA followed by Tukey post-hoc tests. BP, blood pressure; Sema, semaglutide; Veh, vehicle.
